# Benign Cystic Mesothelioma: A Rare Cause for Scrotal Swelling

**DOI:** 10.1155/2012/572186

**Published:** 2012-05-23

**Authors:** A. Aber, A. Tahir, V. Arumuham, G. Smith, S. Almpanis

**Affiliations:** ^1^Urology Department, Royal Free Hampstead NHS Trust, Pond Street, London NW3 2QG, UK; ^2^Urology Department, Homerton Hospital, Homerton Row, London E9 6SR, UK

## Abstract

Benign cystic mesothelioma of the tunica vaginalis is a rare occurrence. It usually presents with painless gradual swelling in the scrotum. These types of benign mesotheliomas typically occur in the peritoneum and usually affect young to middle-aged patients. We present in this case an unusual case of benign cystic mesothelioma of the tunica vaginalis in a 77-year-old male patient.

## 1. Case Report

A 77-year-old man presented to the urology outpatient clinic with a right scrotal swelling; the patient did not report any symptoms such as pain or discomfort from the site of the swelling. However, the patient did express his concerns about the slow and gradual growth of the mass in his scrotum for months. The patient had no history of asbestos exposure or any surgical procedures.

**Figure 1:**
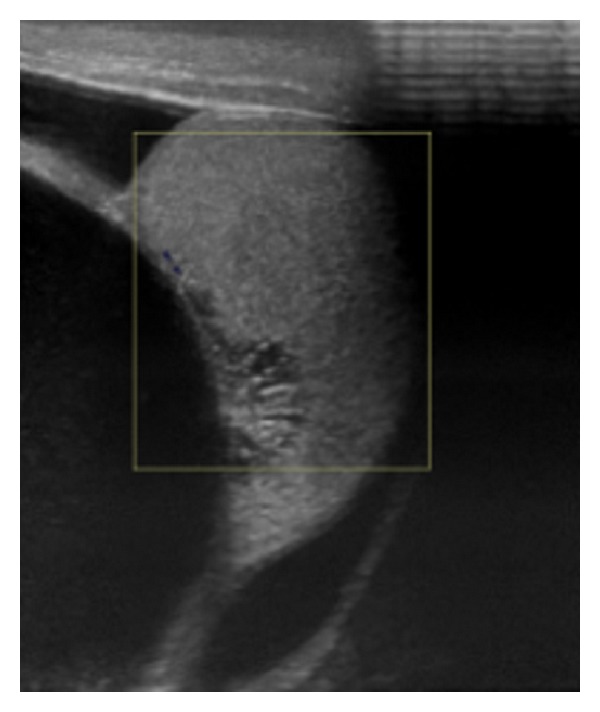
Ultrasound image of the right testicle cystic mass.

On examination he had a tense swelling in the right hemiscrotum, the clinical features of which were consistent with a hydrocele. The scrotal ultrasound scan reported a large 7-8 cm multiloculated hydrocele (Figure 1).

**Figure 2:**
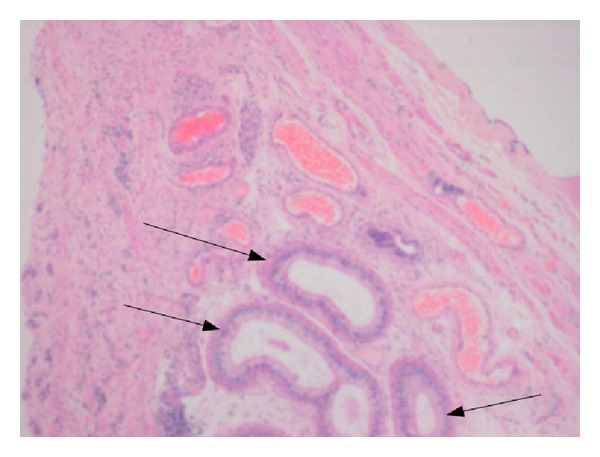
The lining of the mesothelioma cyst showing single layer of cuboidal cells.

Following a thorough review, the patient underwent surgery to excise the right scrotal mass. Surgery revealed a tense multicystic mass which was completely excised and sent for histopathological assessment. The histopathology review of the scrotal cyst did report a benign multicystic mesothelioma arising from the tunica vaginalis (Figure 2).

Four months later the patient presented for post-operative followup, and on examination the right testis was hard and fixed in the hemiscrotum but the right hemiscrotum, returned to the normal size. He was asymptomatic and free of recurrence.

## 2. Discussion

Benign multicystic mesothelioma (BMM), also referred to as cystic mesothelioma or multilocular inclusion cyst, is an intermediate-grade neoplasm of the mesothelial cells. Most cases that have been described involve the abdominal cavity, although few cases involving the spermatic cord and tunica vaginalis have been reported [1, 2]. All the reported cases in the tunica vaginalis did exhibit similar features to BMM in the peritoneum; this could be due to that fact that, during embryonic life, the tunica vaginalis forms from an outpouching of the peritoneal fold [3–6].

It is worth noting that BMM tends to recur locally, making it more aggressive than other benign tumors, which arise from mesothelial cells in the genital tract such as adenomatoid tumour; however, it is more benign than malignant peritoneal mesotheliomas [7]. Unlike malignant mesothelioma, cystic mesothelioma is not associated with prior asbestos exposure [7]. Although the tumor does not metastasize, there is a high recurrence rate that has been reported to be 27%–75% in the 3 months to 19 years after initial resection [8].

BMM more commonly occurs in the peritoneum and the patient population is usually young to middle-aged women [8]. Men are also affected. However, there is a substantial female predominance, as some studies [9] reported 81.2% of cases occurred in women.

 There are no proved risk factors for cystic mesothelioma, but cysts are commonly found at sites of prior surgery or inflammation [1]. BMMs are relatively rare tumors that arise from the serosal surface of the pleura, peritoneum, and pericardium. On rare occasions, they originate from the tunica vaginalis of the testis in which case they manifest as a paratesticular mass.

The present case presents a unique case of this benign tumour in an elderly patient; the histology as well as the radiological features exhibits a similar pattern as the peritoneal BMM. Because of its rarity most urologists might not be familiar with its macroscopic and radiological features. However, especially with its distinct multicystic nature it is worth considering it as part of the differential diagnoses for a scrotal multicystic swelling.
